# Theta-Burst Stimulation for Cognitive Enhancement in Parkinson's Disease With Mild Cognitive Impairment: A Randomized, Double-Blind, Sham-Controlled Trial

**DOI:** 10.3389/fneur.2020.584374

**Published:** 2020-12-21

**Authors:** Stefan Lang, Liu Shi Gan, Eun Jin Yoon, Alexandru Hanganu, Mekale Kibreab, Jenelle Cheetham, Tracy Hammer, Iris Kathol, Justyna Sarna, Davide Martino, Oury Monchi

**Affiliations:** ^1^Cumming School of Medicine, Hotchkiss Brain Institute, Calgary, AB, Canada; ^2^Department of Clinical Neurosciences, University of Calgary, Calgary, AB, Canada; ^3^Non-invasive Neurostimulation Network, University of Calgary, Calgary, AB, Canada; ^4^Institut Universitaire de Gériatrie de Montréal, Centre de Recherche, Montréal, QC, Canada; ^5^Department of Radiology, University of Calgary, Calgary, AB, Canada

**Keywords:** Parkinson's disease, cognition, transcranial magnetic stimulation, functional connectivity, theta-burst stimulation

## Abstract

**Background:** Mild cognitive impairment is a common non-motor symptom of Parkinson's disease (PD-MCI) and has minimal treatment options.

**Objective:** In this double-blind, randomized, sham-controlled trial, we assessed the effect of repeated sessions of intermittent theta-burst stimulation over the left dorsolateral prefrontal cortex on cognition and brain connectivity in subjects with PD-MCI.

**Methods:** Forty-one subjects were randomized to receive real (*n* = 21) or sham stimulation (*n* = 20). All subjects underwent neuropsychological assessments before, 1 day, and 1 month after stimulation. Subjects also underwent resting-state functional magnetic resonance imaging before and 48 h after stimulation. The primary outcome was the change in the cognitive domain (executive function, attention, memory, language, and visuospatial abilities) z-scores across time.

**Results:** There was an insignificant effect on cognitive domain z-scores across time when comparing real with sham stimulation and correcting for multiple comparisons across cognitive domains (*p* > 0.05 Bonferroni correction). However, the real stimulation group demonstrated a trend toward improved executive functioning scores at the 1-month follow-up compared with sham (*p* < 0.05 uncorrected). After real stimulation, the connectivity of the stimulation site showed decreased connectivity to the left caudate head. There was no change in connectivity within or between the stimulation network (a network of cortical regions connected to the stimulation site) and the striatal network. However, higher baseline connectivity between the stimulation network and the striatal network was associated with improved executive function scores at 1 month.

**Conclusions:** These results suggest that intermittent theta-burst stimulation over the dorsolateral prefrontal cortex in subjects with PD-MCI has minimal effect on cognition compared with sham, although there were trends toward improved executive function. This intervention may be more effective in subjects with higher baseline connectivity between the stimulation network and the striatal network. This trial supports further investigation focusing on executive function and incorporating connectivity-based targeting.

**Clinical Trial Registration:** www.ClinicalTrials.gov, identifier NCT03243214.

## Introduction

Parkinson's disease is a common neurodegenerative disease that frequently results in cognitive impairment and dementia (1, 2). A growing body of literature supports the idea that cognitive decline in Parkinson's disease is mediated by degeneration and dysfunction of neural networks (3). Therefore, therapies designed to target neural networks may provide an opportunity to improve cognitive symptoms. Transcranial magnetic stimulation (TMS) can modulate activity and connectivity within neural networks (4–6) and has shown promise in improving cognitive abilities in healthy subjects (7–9), including in healthy older adults (10), subjects with mild cognitive impairment and Alzheimer's disease (11), and depressed Parkinson's disease subjects (12). However, lack of an effect in cognitively normal PD after a single stimulation session has also been reported (13).

Recent work assessing the efficacy of TMS in Parkinson's disease with mild cognitive impairment (PD-MCI) has shown mixed results. In the first study, a 2-week course of bilateral repetitive TMS (rTMS) at 20 Hz, or sham stimulation, was directed over the dorsolateral prefrontal cortex (DLPFC) (14). Cognition was assessed on the same day as the last stimulation session. No significant group differences were observed for their primary outcome: the total score on the Dementia Rating Scale II. The second study investigated the effects of intermittent theta-burst stimulation (iTBS) over the left DLPFC (15). iTBS is a patterned form of TMS (16) that can be administered in less time than rTMS, may facilitate induction of plasticity mechanisms (17), and may have a beneficial effect on executive functions in healthy subjects (9). Subjects had six iTBS sessions for 6 days. Overall, there were no significant group differences in cognition, which was defined as the average z-score resulting from a neuropsychological battery assessing five cognitive domains. However, the real stimulation group experienced a significant increase in global cognition, attention, and visuospatial abilities, whereas the sham group experienced an increase in attention alone. Notably, this improvement in the real group was seen at a follow-up of 1 month. Overall, repeated sessions of iTBS over the left DLPFC appears more promising than bilateral rTMS [or single session iTBS (13)].

Neither trial assessed for changes in brain connectivity. A recent meta-analysis on the effects of rTMS on resting-state connectivity (18) demonstrated that changes could not be summarized into a consistent effect across studies. This is likely due to the vast stimulation parameter space, the diverse patient groups, and the complex relationship between stimulation and brain state (19). Nevertheless, rTMS of the left DLPFC has been shown to increase the amount of dopamine in the ipsilateral caudate head (20), as well as cause widespread changes in brain networks (18, 21, 22), including alteration of frontostriatal connectivity (23, 24). Importantly, cognitive impairment in Parkinson's disease has a substantial “dysexecutive” component, which has been linked to dopaminergic-dependant frontostriatal networks (25). Therefore, we hypothesized that targeting a region of the DLPFC previously implicated in executive dysfunction in PD-MCI (26), with the goal of increasing dopamine release into the ipsilateral caudate and modulating frontostriatal connectivity, may improve cognitive abilities in PD-MCI.

Thus, the objective of the current investigation was 2-fold: first, to attempt to replicate the promising results applying iTBS to the left DLPFC in PD-MCI using a more rigorous trial design (preregistered, randomized, with allocation concealment); and secondly, to investigate the effect of stimulation on functional connectivity, focusing on the DLPFC and ipsilateral striatum. The left DLPFC target was chosen based on the previous implication of this region in executive deficits in PD-MCI (26). We hypothesized we would see an improvement in cognitive ability in the real group when compared with the sham group, at a follow-up of 1 month, as in the previously described trial (15). With respect to the imaging analysis, we hypothesized frontostriatal connectivity would be altered after real stimulation. We tested this with a focused analysis assessing connectivity specifically between the stimulation site and the left caudate head, along with other striatal subregions. However, given the increasing literature showing network-level effects of stimulation (18), we also assessed for changes in connectivity from a network and whole-brain perspective. Finally, we explored whether any baseline demographic or imaging features might help predict stimulation response in the real group.

## Methods

### Subjects

**Figure 1:**
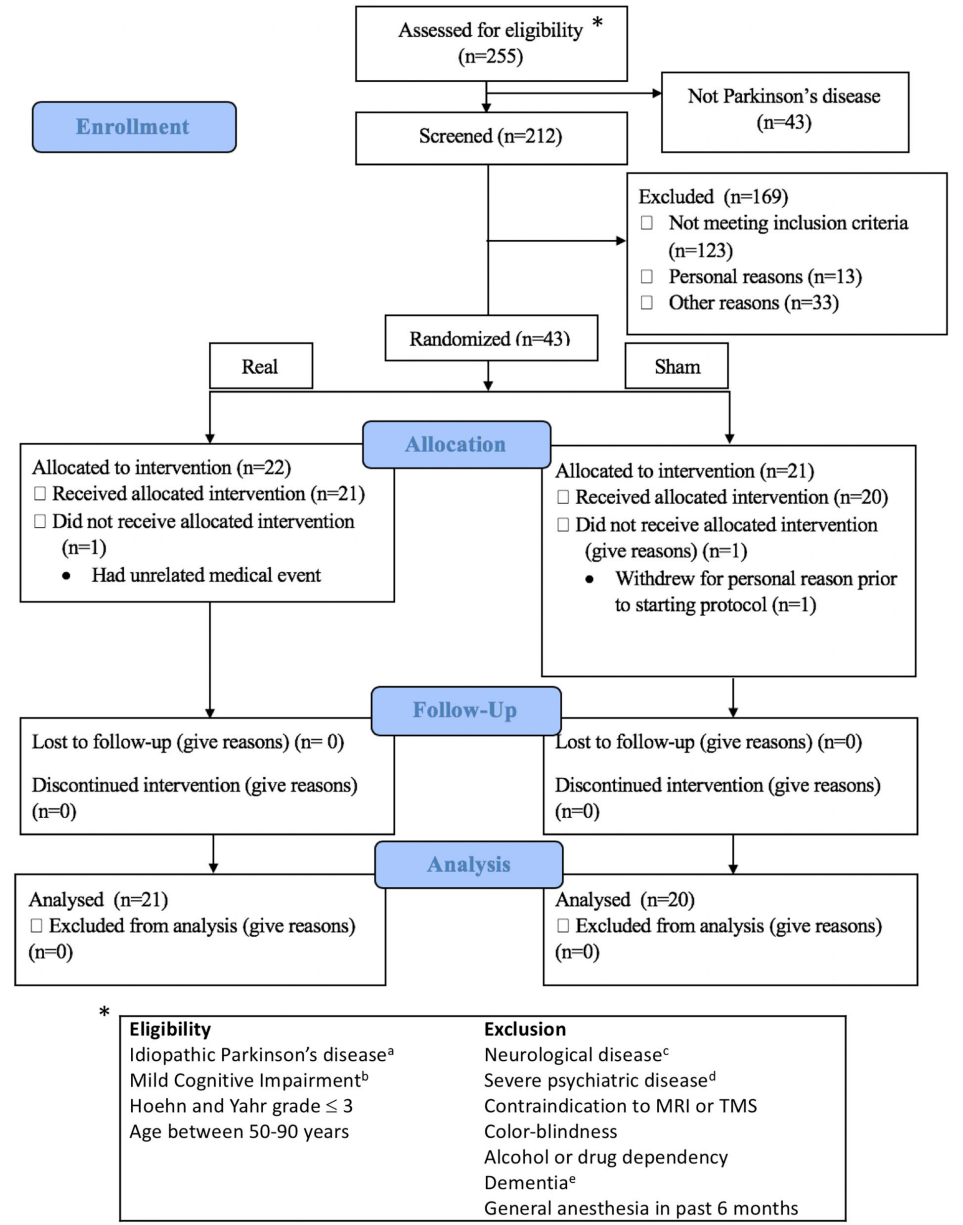
Consort flow diagram and eligibility criteria. Exclusion details in Supplementary Methods I. ^a^Diagnosed by a movement disorder neurologist; ^b^Assessed according to MDS Task Force Level II criteria; ^c^Other than idiopathic Parkinson's disease; ^d^Documented in the subjects' clinical records by a physician; ^e^Severe cognitive impairment affecting activities of daily living. *indicates to the eligibility criteria.

From September 2016 to February 2020, 212 subjects with Parkinson's disease were assessed for eligibility (Figure 1), and 41 subjects (21 real, 20 sham) completed the study. All subjects provided informed consent. The University of Calgary Research Ethics Board approved the protocol. Subjects continued on their routine medication schedule for the entirety of the trial. One subject (sham group) was taking an anti-cholinesterase medication.

### Trial Overview

This study was a randomized, double-blind, sham-controlled, parallel-group trial conducted in accordance with CONSORT guidelines and preregistered to ClinicalTrials.gov (identifier NCT03243214). We estimated that a sample size of 32 participants per group would provide 80% power at a significance level of 0.05 to detect a 0.25 between-group difference in the change of the average z-score of an individual cognitive domain. However, this sample size was not reached due to recruitment difficulty. Despite this, the sample size was consistent with the aforementioned previous trials investigating non-invasive brain stimulation in PD-MCI.

**Figure 2:**
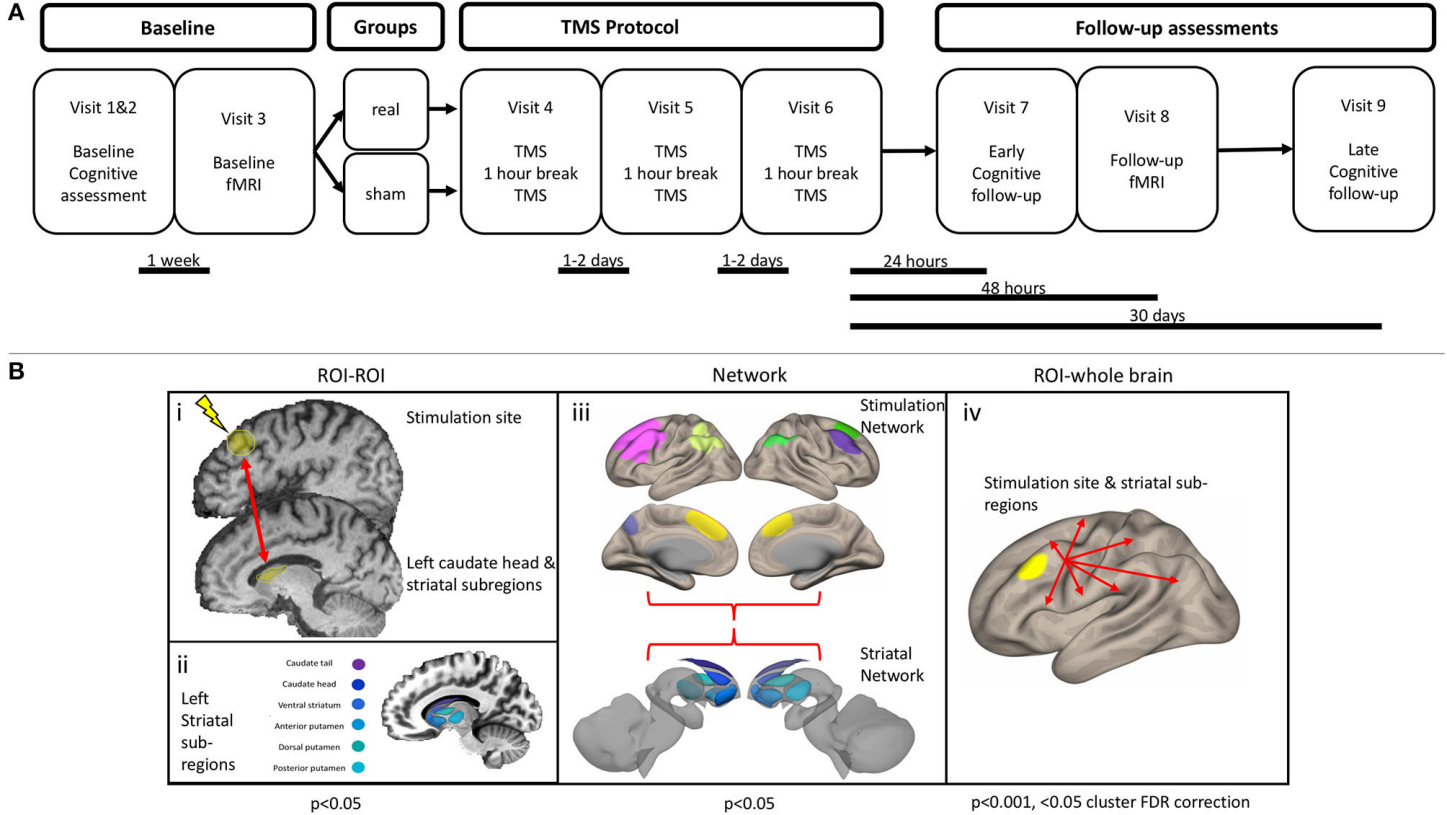
**(A)** Study protocol. **(B)** Functional connectivity analysis outline. (i) Region of interest (ROI) to ROI analysis. An ROI–ROI analysis was performed, specifically assessing the connectivity between the stimulation site and the left caudate head, along with five other striatal subregions. (ii) Left striatal subregions were obtained from a functional parcellation of the striatum (28). (iii) Connectivity within and between the striatal network and stimulation network was assessed. Striatal network consisted of the six subregions of the left striatum. Stimulation network consisted of seven cortical ROIs, which display strong functional connectivity with the stimulation site. (iv) An ROI–whole brain analysis was performed with the stimulation site and each of the six left striatal subregions.

Participants were diagnosed by movement disorder neurologists and met the UK Brain Bank Society criteria for idiopathic Parkinson's disease (27). Participants were block-randomized according to sex and age (50–65 or >65 years), with a block size of four. Once assigned to a block, subjects were randomized on a 1:1 basis into real or sham stimulation. Randomization was accomplished with a random number generator and was performed by an independent study investigator who was not involved in TMS administration, outcome assessment, or data analysis. Investigators performing TMS were not made aware of the randomization until immediately before the first session. The outcome assessment was performed by a psychometrist who was fully blinded to treatment assignment. Subjects were involved in a total of nine study visits. The first two visits consisted of a baseline assessment of cognition with a full neuropsychological battery, additional questionnaires, and the Unified Parkinson's disease rating scale part III (UPDRS-III). The results were used to determine eligibility for the trial (if meeting the MCI requirements) and as a baseline measure of cognition. If eligible, subjects came back within 1 week to perform the first of two MRI scans. After the initial MRI scan, subjects returned for three visits (for 1 week) to participate in the stimulation sessions (described in more detail later). Twenty-four hours after the final stimulation session, subjects returned for a sixth visit, where they underwent an early neuropsychological assessment follow-up. Forty-eight hours after the final stimulation session, subjects returned for the follow-up MRI scan. This gap between the early neuropsychological follow-up and the MRI scan was intended to minimize fatigue. Finally, subjects returned 1 month after the final stimulation session for a delayed assessment of cognition. Details of the study protocol can be found in Figure 2A. The only deviation from the preplanned study protocol was the removal of a 1-week neuropsychological follow-up. The resting-state functional magnetic resonance imaging (fMRI) analysis was not included in the registered protocol. Complete reasons for subject exclusion are shown in Supplementary Table 1.

The primary outcome was the change in cognitive domain z-scores across time, between real and sham. We hypothesized that real stimulation would improve cognition over time as compared with sham stimulation. Secondary outcome measures included motor and behavior scores, measured by the UPDRS-III and the Beck Depression Inventory-II/Beck Anxiety Inventory (BDI-II/BAI).

### Neuropsychological Assessment

At baseline, each subject underwent a comprehensive neuropsychological evaluation with a total of 17 cognitive measures to assess five cognitive domains (attention, executive functioning, language, memory, and visuospatial ability) (Supplementary Table 2). Each test was administered and scored by a blinded psychometrist, and the raw scores were converted to z-scores based on age and (where appropriate) education and/or sex-normed data. Subjects were classified as having MCI if they met the Movement Disorder Task Force Level II criteria for MCI in Parkinson's disease (29). These requirements were as follows: (1) performance > 1.0 standard deviation below the standardized mean on at least two tests within or across cognitive domains; (2) subjective complaint of cognitive decline by patient or accompanying person; (3) absence of a significant decline in daily living activities; (4) absence of dementia. Subsequently, domain-specific average z-scores were calculated by averaging each individual test within each domain. Global cognition was defined as the average of the domain-specific z-scores. After stimulation, subjects again underwent an early (24 h) and late (1 month) cognitive assessment. In these instances, alternative forms (for most cognitive measures) were administered to minimize the learning effect. At each follow-up, subjects also completed the BDI-II, the BAI, and the UPDRS-III. The outcome assessor was fully blinded to the treatment group.

### Stimulation Sessions

Subjects received a total of six iTBS sessions for 1 week. Real stimulation was administered at 80% of the active motor threshold over the left DLPFC (15, 16). Stimulation was carried out using the MagStim Super Rapid^2^ magnetic stimulator (The Magstim Company Ltd, UK) and the air-cooled figure-of-eight coil (AirFilm Coil, The Magstim Company Ltd, UK). For the exact localization of cortical targets, we used the frameless stereotaxic neuronavigation system implemented by the Brainsight software (Rogue Research, Canada). Each subject's high-resolution structural scan was loaded into the software and normalized to MNI space. The left DLPFC was identified using group coordinates from a previous fMRI study implicating this region in the executive deficits of PD-MCI (MNI coordinates: −48, 26, 36) (26). The region was slightly adjusted on a subject-specific level to ensure stimulation occurred directly over the cortex and not over a sulcus. Stimulation intensity was adjusted to reflect 80% of the active motor threshold (aMT), which was determined through the following procedure (15, 16). Motor evoked potentials were recorded from the right first dorsal interosseous muscle using AgCl surface electrodes (Kendall H69P Cloth Electrodes, Covidien, Medtronic, USA) and the Brainsight electromyography system. The active electrode was placed over the muscle belly and the reference electrode over the metacarpophalangeal joint of the index finger. The coil was placed tangentially to the scalp at a 45°angle from the midline of the central sulcus, inducing a posterior–anterior current flow in the brain. The optimal position for stimulating the left cortical motor area of the right hand was found by moving the coil in small steps around the presumed cortical area while observing the activation level of the right first dorsal interosseous muscle. Visual feedback of the electromyography activity was provided on a computer screen, and the participants were asked to lightly contract their thumb and index to maintain 20% of their maximum voluntary contraction. The aMT was determined by applying single TMS pulses over the left motor hand area and increasing the intensity until a motor evoked potential was reliably elicited (>200 uV) for at least 50% of 10 consecutive stimulations, and lesser levels of stimulation failed to elicit consistent muscle contractions (30). The aMT was assessed at the start of each stimulation day. After the determination of the aMT, iTBS was administered at 80% aMT over the DLPFC. Three pulses delivered at a frequency of 50 Hz and repeated with a frequency of 5 Hz were given for 2 s. This was repeated every 10 s for a total of 3 min (for 600 pulses) (16). If the participant reported discomfort during stimulation, the intensity was lowered slightly before continuing. This occurred on four occasions (Supplementary Table 3). Sham stimulation was performed with the sham AirFilm Coil, keeping everything else the same. The sham coil produced clicking sounds and slight sensory stimulation while producing nearly no electric field at the center (31). To assess for blinding, after the last stimulation session, subjects completed the Stanford Expectation of Treatment Scale. As part of this scale, subjects were directly asked which condition they thought they received and what (if any) benefit they felt.

### Magnetic Resonance Imaging

All subjects underwent two MRI scans, one at baseline and one 48 h after the final stimulation session.

### Image Acquisition

Subjects were scanned at the Seaman Family MR Center, at the University of Calgary, with a 3T GE Discovery MR750 scanner. Sessions included a high-resolution, T1-weighted, 3D volume acquisition for anatomic localization (repetition time = 7.18 ms, echo time = 2.25 ms, flip angle 10°, voxel size 1mm^3^, 172 slices), followed by echo-planar T2*-weighted image acquisitions with blood oxygen level-dependent (BOLD) contrast (repetition time = 2.9 s, echo time = 30 ms; flip angle, 90°, voxel size 2.5 × 2.5 × 3 mm, 48 slices, 152 volumes). Resting-state fMRI was acquired over one 7.34-min run in a single session. During the scan, participants were presented with a black fixation cross on a white background and were instructed to keep their eyes open and look at the cross without falling asleep.

### Image Preprocessing

Raw and temporal signal to noise images were visually examined for the presence of MRI artifact. No subjects had significant artifact necessitating exclusion. Images were preprocessed using SPM 12 (32). Functional images underwent realignment and unwarping as well as slice-time correction. The high-resolution structural images were co-registered to the mean functional image. Visual inspection for quality control was performed at each stage. The co-registered structural images were segmented into gray matter, white matter, and cerebrospinal fluid (CSF), and non-linearly normalized into MNI space using SPM's unified segmentation (33). Images were spatially resampled at 2 mm^3^ before analysis.

### Image Denoising

Denoising of the functional data was performed using the MATLAB toolbox Conn (34). Physiological and other sources of noise from the white matter and CSF signal were estimated using the aCompcor method (35, 36). Five principal components were extracted from eroded CSF and white matter masks and included as covariates of no-interest. To account for motion, movement parameters, and their first temporal derivative, were also included in the regression. Further quality assurance to detect outliers in motion and global signal intensity change was performed. Volumes with >3-mm change of maximal composite motion, or a (BOLD) change > 3 SD from the mean, were flagged and included as regressors in the first-level analysis. Subjects with >50% of volumes flagged for artifact were removed from further MRI analysis. Linear detrending, to remove signal drift, was performed. The residual BOLD time series was subjected to a high-pass filter (>0.008 Hz) before calculating resting-state connectivity. A full bandpass filter (i.e., 0.008–0.1 Hz) was not used, as there is accumulating evidence for the relevance of higher frequencies in the resting state signal (37).

### Functional Connectivity Analysis

We performed three analyses at different levels of spatial resolution [region of interest (ROI)–ROI, network, and ROI–whole brain].

#### Region of Interest–Region of Interest

Given previous literature showing an increase of dopamine specifically within the caudate head after rTMS, we were primarily interested in assessing the change in connectivity of this structure with the stimulation site. For the stimulation site, we placed a 10-mm spherical ROI centered over the stimulation coordinates, whereas the left caudate head was defined using an ROI from a functional parcellation (28) (Figure 2Bi). To explore the specificity of any changes, we also assessed connectivity between the stimulation site and five other striatal subregions, using the same functional parcellation (Figure 2Bii). The residual BOLD time-course was averaged within each ROI, and functional connectivity was calculated as the z-transformed Pearson correlation coefficient.

#### Network

The stimulation network was defined as a network of cortical areas exhibiting high connectivity with the stimulation site (Figure 2Biii, Supplementary Methods I). The striatal network consisted of the six left striatal subregions. Within and between network connectivity was calculated with the *withinbetweenROI_test* function in Conn, using the z-transformed Pearson correlation coefficients. This function calculates the average connectivity between all ROIs within and between the two networks.

#### Region of Interest-Whole Brain

To explore connectivity changes more broadly, we calculated seed-based connectivity across the whole brain (Figure 2Biv). This was performed by calculating the functional connectivity between each ROI time-course and the time-course of every voxel.

### Predictor of Response

Baseline connectivity profiles were used in the exploratory analysis assessing predictors of cognitive change. We focused on the connectivity between the stimulation site and left caudate head and connectivity between the stimulation network and striatal network.

### Statistics

Statistical analyses of the clinical data were performed in MATLAB (MathWorks®, MA, USA). Kolmogorov–Smirnov tests were used to assess for normality. Demographic variables were compared between groups with two-sample *t*-tests, chi-squared tests (for categorical data), or Mann–Whitney *U* tests for non-normally distributed data. To formally test for blinding, a Fisher exact test was utilized. We assessed for equal proportions of subjects in each treatment arm who responded with “Active/TMS” or “Sham/Unknown” when asked which type of stimulation they received, as well as those who felt they received some benefit or not after treatment.

To assess for changes in cognitive performance across time, we utilized linear mixed effect models with fixed effects of time (baseline, early, late) and group (real, sham) with a random effect of the subject. This was performed with MATLAB's *fitlme* function, which estimates parameters of the model using maximum likelihood estimation. The primary outcome was defined as the interaction term between time and condition. The significance of model coefficients was set at *p* < 0.01, controlling for multiple comparisons across five cognitive domains. We considered an uncorrected *p*-value of <0.05 as a trend of interest. Because all cognitive scores were normally distributed, *post hoc* testing of significant coefficients was performed with paired sample *t*-tests. Separate models were used for each cognitive domain. We also assessed global cognition, which represented the average score across all five domains. Secondary outcomes (BDI-II, BAI, and UPDRS-III) were assessed in the same manner. In a supplementary analysis (Supplementary Methods II), we assessed whether baseline global cognition or baseline executive function was related to any change in cognitive scores within the real stimulation group. We also assessed whether a more stringent classification of MCI would change our results.

Statistical analyses of the resting-state fMRI data were performed within the Conn software. The change in connectivity between groups across time was assessed with a repeated measure two-way ANOVA, with a primary focus on the condition*time interaction contrast. All group comparisons were adjusted for average motion, the number of invalid scans, and baseline visuospatial ability. For the ROI-ROI and network analysis, a threshold of *p* < 0.05 was implemented. For the seed-whole brain analysis, significant clusters were defined with a height threshold of *p* < 0.001 (two-tailed, uncorrected), followed by a cluster threshold of *p* < 0.05 with a false discovery rate correction for multiple comparisons. Significant effects were correlated with significant changes in cognition in the real group. Lastly, we performed an exploratory analysis looking for demographic and imaging variables that may help predict the response to stimulation. We did this by assessing the relationship between baseline demographics (age, UPDRS, BDI-II, BAI, and MoCA) and baseline imaging features (stimulation site–left caudate head and stimulation network–striatal network) with any changes in cognition in the real stimulation group. This was performed with a stepwise linear regression model using MATLABS's *stepwiselm* function. This function uses forward and backward regression to determine a final model, where at each step terms are included or removed based on the *p*-value for an F test of the change in the sum of squared error. The criteria to enter the model was set at 0.05, whereas the criteria to be removed from the model was set at 0.10. The final model was determined to be significant if the *p*-value was less than 0.05.

## Results

### Demographics

There was no difference at baseline in demographic variables (Table 1). One subject in the real group was excluded from the fMRI analysis due to >50% of the fMRI volumes being flagged for an artifact. Therefore, 40 subjects (20 real, 20 sham) had satisfactory fMRI data. After the exclusion of this subject, the real group had less movement and fewer volumes flagged for an artifact (Table 1). Hence, both of these quality control metrics were used as covariates in all subsequent MRI analyses assessing group differences. There were no study-related serious adverse events (Supplementary Table 3).

**Table 1:** Demographics.

**Demographics (baseline)**	**Real [mean (SD)]**	**Sham [mean (SD)]**	**Test statistic**	***P*-value**
N	21	20		
Age	68.43 (8.4)	68.76 (8.3)	*t* = 0.127	0.900
Sex (M:F)	14:7	13:7	*x*^2^ = 0.013	0.910
Education (years)	12.9 (2.6)	13.7 (2.3)	*t* = 0.953	0.347
UPDRS-III	20.7 (10.2)	23.5 (13.2)	*t* = 0.748	0.459
LED (mg/day)	911.95 (522.2)	930.10 (396.2)	*t* = 0.125	0.901
Disease duration (years)	5.95 (4.8)	4.80 (4.0)	*z* = −1.03	0.303
MoCA	22.95 (3.6)	22.90 (4.8)	*t* = −0.040	0.969
BDI-II	12.33 (7.9)	11.05 (6.5)	*t* = −0.566	0.575
BAI	13.10 (9.0)	12.75 (6.6)	*z* = 0.196	0.845
Mean movement (mm/TR)	0.169 (0.06)	0.237 (0.11)	*t* = 2.38	0.02
Excluded volumes (%)	2.1% (3.9%)	7.7% (8.9%)	*z* = 2.88	0.004

*SD, standard deviation; UPDRS-III, Unified Parkinson's disease Rating Scale III; LED, levodopa equivalent dosage; MoCA, Montreal cognitive assessment; BDI-II, Becks Depression Inventory II; BAI, Becks Anxiety Inventory*.

### Blinding

The Stanford Expectation of Treatment survey was administered immediately after the final stimulation session. Data were not recorded for six subjects, leaving a total of 35 with complete data (real = 19; sham = 16). Every subject, except for one individual in the real stimulation condition, stated they felt they received active stimulation (Fisher exact: *p* = 1.0). We also assessed whether subjects felt they received any subjective benefit after the stimulation sessions. This was coded as a Yes/No response. There was no difference in the proportion of subjects across treatment arms who felt they received some benefit (37% sham, 42% real; Fisher exact: *p* = 0.510).

### Cognition

All cognitive scores (mean and standard deviation) for each group and time point are displayed in Supplementary Table 4.

#### Global Cognition

**Figure 3:**
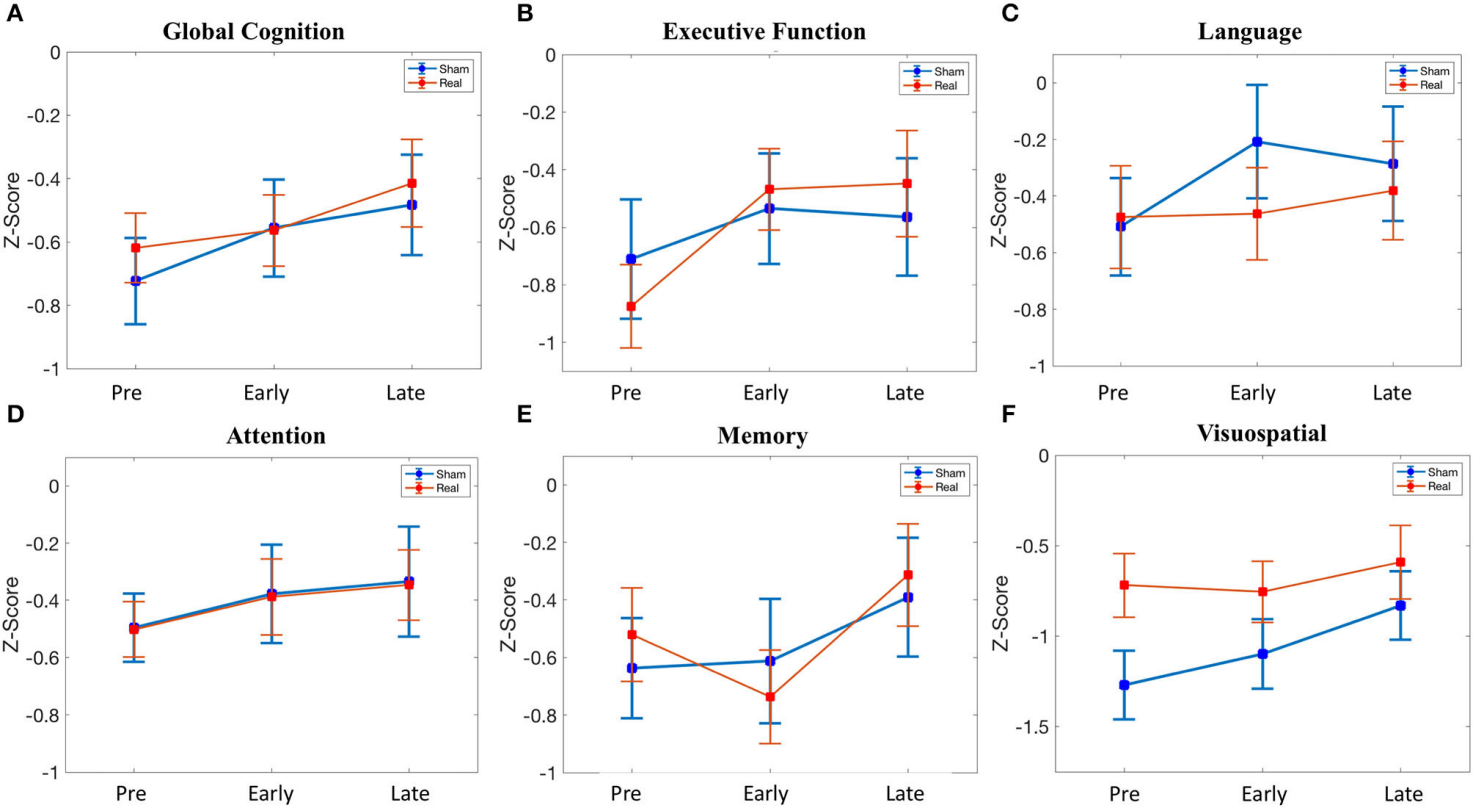
Cognitive scores across time (pre-stimulation, early (24 h after stimulation), late (1 month after stimulation). **(A)** Global cognition. **(B)** Executive function. **(C)** Language. **(D)** Attention. **(E)** Memory. **(F)** Visuospatial. See Supplementary Table 4 for details.

There was no difference in global cognition at baseline [*t*_(39)_ = 0.60, *p* = 0.551]. In the linear mixed effect model, there was no main effect of condition [β = 0.105, 95% CI = −0.265–0.475, *t*_(117)_ = 0.56, *p* = 0.576], whereas there was an effect of the early follow-up [β = 0.168, 95% CI = 0.054–0.282, *t*_(117)_ = 2.91, *p* = 0.004] and the late follow-up [β = 0.241, 95% CI = 0.126–0.355, *t*_(117)_ = 4.16, *p* = 0.00006]. *Post hoc* testing demonstrated that the effect of the early follow-up was driven by an increase in the sham condition [*t*_(19)_ = 3.19, *p* = 0.0049], with no change in the real condition, whereas the effect of the late follow-up was driven by an increase in both sham [*t*_(19)_ = 3.37, *p* = 0.003] and real [*t*_(20)_ = 3.84, *p* = 0.001] condition. Despite the effect of both early and late follow-up, there was no condition*time interaction [early*condition: β = −0.113, 95% CI = −0.272–0.047, *t*_(117)_ = −1.40, *p* = 0.165; late*condition: β = −0.036, 95% CI = −0.196–0.124, *t*_(117)_ = −0.449, *p* = 0.655] (Figure 3A).

#### Executive Function

There was no difference in executive function at baseline [*t*_(39)_ = −0.66, *p* = 0.515]. In the linear mixed effect model, there was no main effect of condition [β = −0.165, 95% CI = −0.657–0.327, *t*_(117)_ = −0.663, *p* = 0.509], early follow-up [β = 0.175, 95% CI = −0.019–0.369, *t*_(117)_ = 1.79, *p* = 0.07], or late follow-up [β = 0.146, 95% CI = −0.048–0.340, *t*_(117)_ = 1.49, *p* = 0.139]. There was no condition*time interaction for the early follow-up [β = 0.233, 95% CI = −0.038–0.504, *t*_(117)_ = 1.70, *p* = 0.092], but there was a trend for the late follow-up [β = 0.281, 95% CI = 0.010–0.552, *t*_(117)_ = 2.05, *p* = 0.042]. Given this trend (*p* < 0.05 uncorrected) in the condition*time interaction at the late follow-up, we performed *post hoc* testing. This revealed an increase in executive function at 1 month in the real group [*t*_(20)_ = 3.86, *p* = 0.00097], whereas no change was observed in the sham group (Figure 3B). Baseline global cognitive score, as well as baseline executive function score, was not related to the change in executive function at the delayed time point in the real group (Supplementary Methods II).

#### Language

There was no difference in language at baseline [*t*_(39)_ = 0.134, *p* = 0.894]. In the linear mixed effect model, there was no main effect of condition [β = 0.034, 95% CI = −0.464–0.532, *t*_(117)_ = 0.134, *p* = 0.894]. There was a significant effect of the early follow-up [β = 0.301, 95% CI = 0.078–0.523, *t*_(117)_ = 2.67, *p* = 0.0086] but not the late follow-up [β = 0.223, 95% CI = −0.0002–0.445, *t*_(117)_ = 1.98, *p* = 0.0502]. The effect of the early follow-up was driven by an increase in sham [*t*_(19)_ = 3.21, *p* = 0.0047], whereas there was no change in the real condition. There was no significant condition*time interaction for either the early follow-up [β = −0.289, 95% CI = −0.600–0.022, *t*_(117)_ = −1.84, *p* = 0.068] or the late follow-up [β = −0.129, 95% CI = −0.440–0.182, *t*_(117)_ = −0.819, *p* = 0.414] (Figure 3C).

#### Attention

There was no baseline, main, or interaction effect for attention (Figure 3D).

#### Memory

There was no baseline, main, or interaction effect for memory (Figure 3E).

#### Visuospatial

Despite randomization, there was a difference in visuospatial ability at baseline [*t*_(39)_ = 2.13 *p* = 0.040], although this did not meet significance with multiple comparison correction. There was no significant main effect of condition [β = 0.551, 95% CI = 0.041–1.06, *t*_(117)_ = 2.14, *p* = 0.041], although the trend represented the numerically lower baseline scores in the sham group. There was no effect of the early follow-up [β = 0.172, 95% CI = −0.119–0.462, *t*_(117)_ = 1.17, *p* = 0.245], although there was an effect of the late follow-up [β = 0.439, 95% CI = 0.148–0.730, *t*_(117)_ = 2.99, *p* = 0.003]. The effect of the late follow-up was driven by an increase in sham [*t*_(19)_ = 2.75, *p* = 0.013], with no change in the real group. There was no significant condition*time interaction for the early follow-up [β = −0.207, 95% CI = −0.613–0.199, *t*_(117)_ = −1.01, *p* = 0.314] or the late follow-up [β = −0.311, 95% CI = −0.717–0.095, *t*_(117)_ = −1.51, *p* = 0.132] (Figure 3F).

### Secondary Outcomes: Beck Anxiety Inventory, Beck Depression Inventory-II, and Unified Parkinson's Disease Rating Scale Part III

**Figure 4:**
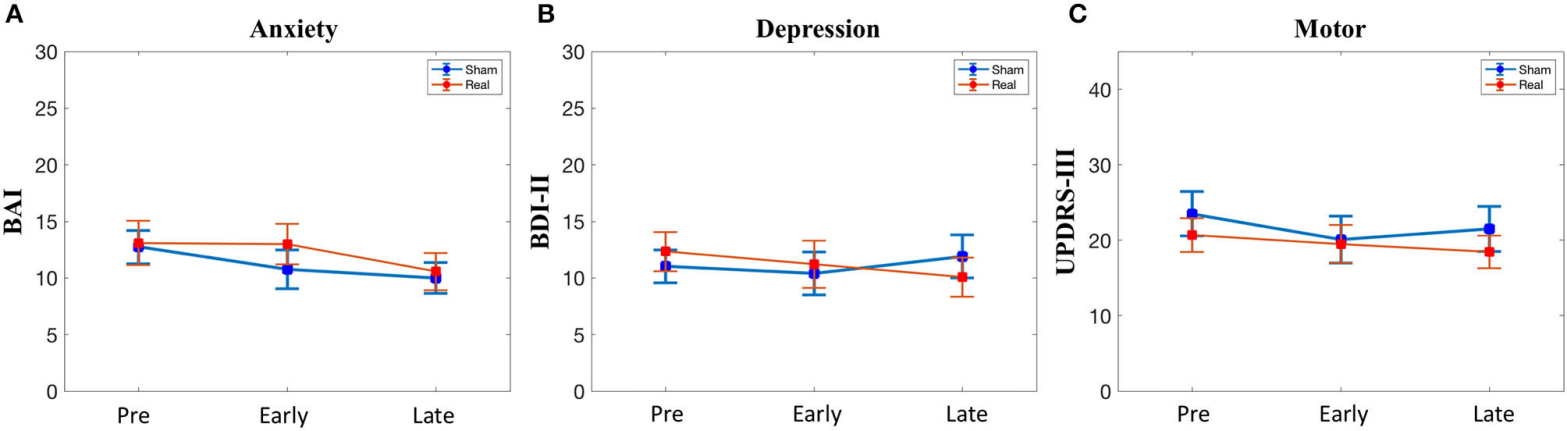
Secondary outcomes. **(A)** Anxiety symptoms. **(B)** Depressive symptoms. **(C)** Motor symptoms. BAI, Beck Anxiety Inventory; BDI, beck depression inventory; UPDRS, unified parkinson disease rating scale part III. No significant condition*time interactions were observed.

There was no difference at baseline in the BAI, BDI-II, or UPDRS-III scores (Table 1). Furthermore, there was no significant condition*time interaction for any of the secondary outcomes (Figure 4).

### Functional Connectivity

#### Region of Interest–Region of Interest

**Figure 5:**
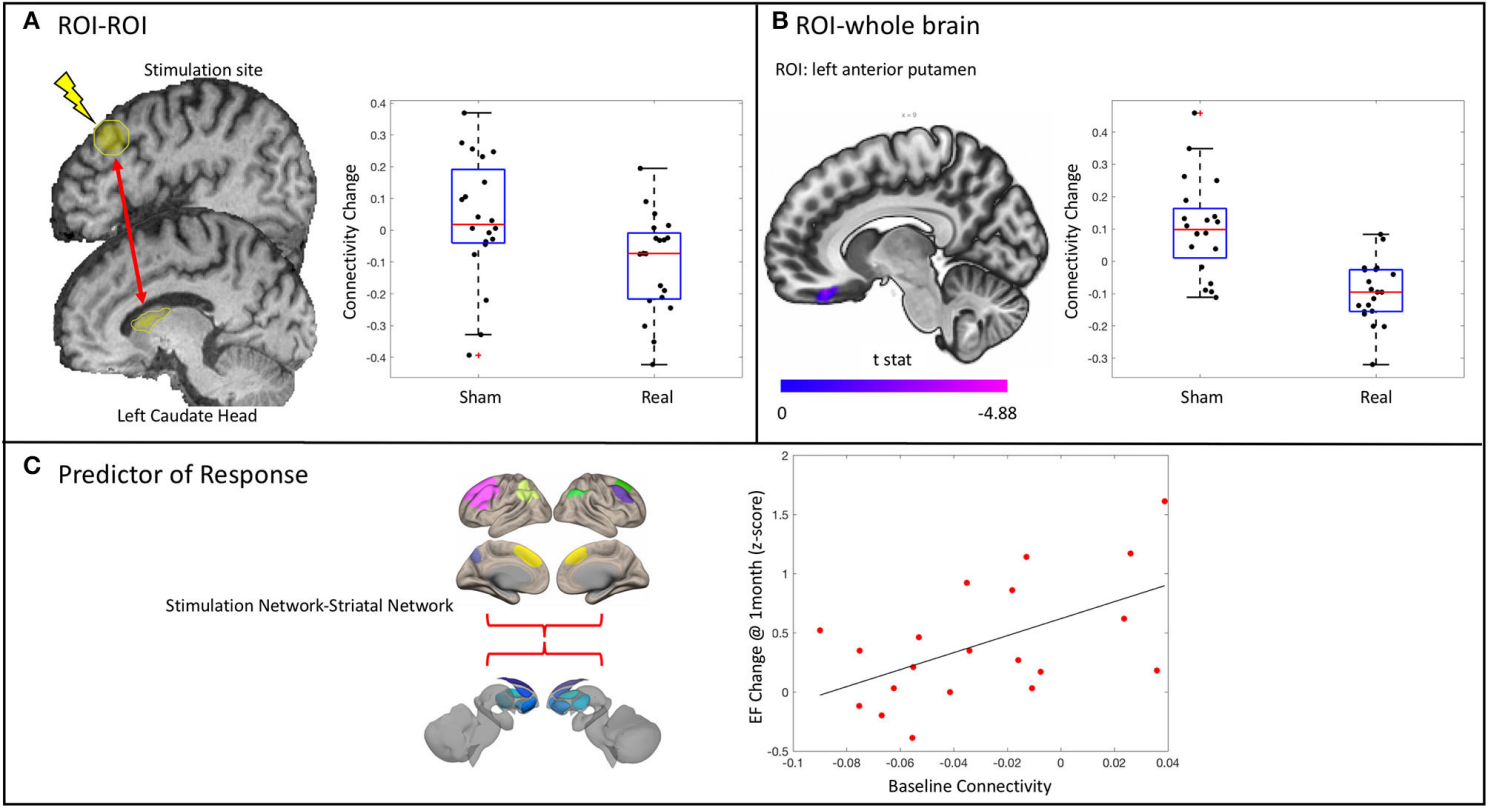
Results from the functional connectivity analysis. **(A)** A significant decrease in connectivity between the stimulation site and the ipsilateral caudate head in the real stimulation group vs. the sham group as revealed by a significant condition*time interaction. Stimulation site did not change connectivity with any of the other striatal subregions assessed. **(B)** ROI–whole brain analysis revealed significantly decreased connectivity between the anterior putamen and the right medial orbitofrontal cortex. **(C)** Baseline connectivity between the stimulation network and the striatal network is related to change in executive functioning (EF) at 1 month in the real stimulation group.

Connectivity between the stimulation site and the left caudate head decreased [*t*_(35)_ = −2.43, *p* = 0.0205] in the real group compared with the sham group (Figure 5A). This was not related to the change in executive functioning in the real group at the delayed time point (r^2^ = 0.126, *p* = 0.1246). There was no effect on connectivity between the stimulation site and any of the other striatal subregions.

#### Network

The change in connectivity within and between both the stimulation network and the left striatal network was not significantly different between groups.

#### Region of Interest–Whole Brain

There was a decrease in connectivity between the left anterior putamen and the right medial orbitofrontal cortex in the real group as compared with the sham group (*p* < 0.001 uncorrected, *p* < 0.05 cluster false discovery rate correction) (Figure 5B). This change was not related to the change in executive function in the real group (*r*^2^ = 0.0034, *p* = 0.8086). There was no difference between groups in the change of connectivity of the stimulation site or any other striatal subregions when assessed across the entire brain.

### Predictors of Response

Given the trend of increased executive functioning at 1 month in the real stimulation group compared with sham, we explored possible demographic and imaging predictors of this response. The overall regression model was significant [*F*_(2, 18)_ = 7.55, *p* = 0.0132] and contained one predictor: the baseline connectivity between the stimulation network and the left striatal network (Figure 5C). No other predictors were significantly related to the change in executive functioning at 1 month in the real group. Notably, the baseline connectivity between the stimulation and striatal network was not related to baseline executive function in the whole group (r^2^ = 0.0158, *p* = 0.44) or in the real group alone (*r*^2^ = 0.00018, *p* = 0.954).

## Discussion

We performed a randomized, double-blind, sham-controlled trial of iTBS applied to the left DLPFC in PD-MCI, assessing longitudinal changes in cognition and brain connectivity. Previous trials assessing the efficacy of TMS in this population have been inconclusive, and none has examined brain connectivity. The main result of the present investigation was that there was no significant difference in cognitive domain scores between real and sham stimulation across time, suggesting the stimulation protocol had a negligible effect on cognition in PD-MCI. However, there was a trend of increased executive functioning in the real vs. the sham group at the 1-month follow-up. There were no changes in any of the secondary outcomes (BDI-II, BAI, or UPDRS-III). Despite this, there was some evidence of altered corticostriatal connectivity 48 h after the last stimulation session.

Despite not reaching significance after multiple comparison correction, the trend of improvement in executive functioning is not unexpected, although inconsistent with previous studies of TMS in PD-MCI. Executive dysfunction in Parkinson's disease is common (38, 39) and results partly from disruption of dopamine-dependant frontostriatal networks (25, 40, 41). For example, fMRI studies of Parkinson's disease subjects have demonstrated that deficits in set-shifting and working memory are related to hypoactivation within frontostriatal loops (42, 43), and this hypoactivation is only present during task phases that normally required coactivation of the striatum (44). Further, dopamine deficiency in the caudate head is strongly correlated with executive dysfunction (45, 46). Importantly, we targeted a region of the DLPFC which has been implicated in executive function deficits in PD-MCI (26), and rTMS of the left DLPFC can increase dopamine binding in the caudate head. Speculatively, this might explain the trend of improved executive functioning observed in the real stimulation group.

However, this effect was observed at 1 month, suggesting that the intervention may have resulted in lasting or delayed changes to frontostriatal networks. Long-lasting effects of repeated stimulation sessions on neuroplasticity markers, such as brain-derived neurotrophic factor, have been seen during investigations of nonhuman primates (47). This implies that plasticity mechanisms might be induced by stimulation, providing a potential mechanism for long-term behavioral effects. Indeed, long-term effects of stimulation have also been noted in depression (48) and post-traumatic stress disorder (49). Supporting the induction of chronic brain changes, we did observe altered frontostriatal connectivity at the follow-up MRI (48 h post-stimulation). Specifically, we observed decreased connectivity between the stimulation site and the left caudate head, although the implication of this is unclear, as the change was not related to clinical improvement. We did not see any change in connectivity within or between the stimulation network or the striatal network, nor did we see any change of the stimulation site when assessed across the entire brain. The seed to the whole-brain investigation revealed a decrease in the connectivity of the left anterior putamen to the right medial orbitofrontal cortex, although again, this was not related to the change in executive functioning. This result can be interpreted in light of previous work showing rTMS of the DLPFC increased dopamine binding within the orbitofrontal cortex (50). This structure has direct anatomical connections to the anterior/ventral putamen (51), perhaps explaining why these regions had altered connectivity after repeated sessions of stimulation. Overall, our results suggest that repeated iTBS of the DLPFC in PD-MCI alters corticostriatal connectivity up to 48 h poststimulation, although it does not specifically alter connectivity within a cortical network of regions functionally connected to the stimulation site. The clinical relevance of these changes remains uncertain. As we did not gather fMRI data at the 1-month time point, we are unable to conclude if the delayed cognitive changes were associated with delayed connectivity changes. However, despite the uncertain relationship between stimulation, connectivity changes, and cognitive changes, we did observe that baseline connectivity between the stimulation network and the striatal network was a predictor of improved executive functioning at 1 month in the real group. This has important implications for future trials and suggests that intact corticostriatal connectivity may be required for a response to stimulation, analogous to what is observed in the depression literature (52). This result could also guide connectivity-based targeting of the DLPFC by implying that selecting a region that results in maximal between network connectivity between its corresponding cortical network and the striatum may result in the greatest benefit.

We classified subjects as having MCI if they performed <1.0 SD below the norm in any two tests across our cognitive battery. Although this is in accordance with the MDS Task Force recommendations, recent studies have suggested using more stringent criteria that may better capture those at risk of further cognitive decline (53, 54). To investigate whether our intervention may preferentially work in those with the more stringent classification of MCI, we repeated our primary analysis using only subjects who met MCI criteria with a cutoff of <1.5 SD in two tests. We did not find any significant effect in this subgroup. Further, we found that worse global cognitive function at baseline was not associated with a beneficial response to stimulation. We also found that worse executive functioning at baseline was not associated with a beneficial response to stimulation. We subtyped our cohort into a common subtype classification of PD-MCI (39, 55) and found that only one subject was classified as single domain non-amnestic (with executive function deficits) (Supplementary Methods II). This precluded an analysis of whether our intervention was more effective in those with a primarily dysexecutive syndrome. It may be possible that stimulation of executive regions or networks will primarily benefit those with a dysexecutive type of syndrome, whereas stimulation of posterior cortical regions may be more beneficial for patients with a posterior cortical subtype. Indeed, work in cognitively normal older adults without PD has demonstrated that stimulation of more posterior cortical regions (parietal regions with high baseline connectivity to the hippocampus) can positively affect memory (56).

Along with altered corticostriatal interactions, PD-MCI has been associated with alterations in distributed brain networks. In general, these findings have been diverse, although several key themes emerge. Firstly, connectivity loss within cortical networks such as the default mode network (DMN) and the frontoparietal/central executive network has been consistently observed (57–59). Further, altered interactions between networks have been associated with cognitive impairment. For example, previous work demonstrated that reduced connectivity between the dorsal attention network and right frontoinsular regions was associated with worse executive and attentional function, whereas increased connectivity of the DMN with occipital regions was associated with worse visuospatial performance (60). Based on these studies, a strategy that explicitly targets cortical networks, with the goal of modulating connectivity or dynamics, may prove beneficial. Our study, in contrast, identified a region of the DLPFC previously implicated in the executive impairment of PD during a task-fMRI study. Importantly, we did not directly target a predefined network based on pre-stimulation imaging. Consequently, along with corticostriatal connectivity, we investigated a “stimulation network” (the set of regions with high-baseline connectivity to the stimulation target). Close examination of the stimulation network demonstrated that it did not directly overlap onto a classical resting-state network (61), but instead, it contained both regions of the central executive (frontal and parietal regions) and DMN (precuneus). This suggests that we stimulated at the interface of these two networks. Supporting this assessment, previous work has shown that depending on the location and orientation of the TMS coil, stimulation of the DLPFC will primarily target either the DMN, the frontoparietal network, or both networks (62). The optimal strategy for choosing a stimulation target remains an open avenue of investigation.

We did not observe any effect on mood or motor outcomes, as measured with the BAI, BDI-II, and the UPDRS-III. rTMS has been used extensively in clinical practice to treat depressive symptomatology in non-PD populations (63). Consistent with our results, decreased DLPFC to ipsilateral caudate connectivity has been observed after rTMS for the treatment of depression (24) [although increased frontostriatal connectivity has also been observed (23)]. This decrease in connectivity was associated with the improvement of depressive symptoms (24). Our protocol did not result in significant decreases in the BDI-II, although we did not recruit patients with severe depression and, therefore, would not be able to detect such changes.

There are several limitations to this study. Most importantly, the trial was underpowered due to recruitment difficulties in a relatively rare subpopulation of PD subjects. Sufficiently powered trials in this population will be difficult without coordination across multiple academic centers. Likely related to this issue, the group difference in executive functioning was not significant at our statistical threshold of *p* < 0.01. As such, we cannot conclude that there was any difference in cognition between groups. However, an effect on executive functioning was the most plausible outcome considering our choice of stimulation site and previous literature in healthy adults (9). Nonetheless, this result was only partly consistent with the previous investigation of iTBS in PD-MCI. Although we did observe the hypothesized improvement at 1 month in the real group (which was significantly different from sham), it was not in the same cognitive domains as the previous trial. We did observe a change in global cognition at 1 month within the real group, although this change was also significant within the sham group. This improvement in both sham and real stimulation may be attributed to the known placebo effect of TMS (64), or it may be due to a learning effect incurred by undergoing repeated assessments (despite using alternative forms). Consistent with a placebo effect, every subject believed they had received real stimulation when asked directly. Also, it is unclear why our investigation failed to replicate the improvement in visuospatial abilities within the real group, although speculatively, this might be due to the significantly higher scores at baseline, possibly resulting in a ceiling effect. Finally, we observed an increase in language and visuospatial abilities in the sham group, which was not observed in the real stimulation group. Although this was not significantly different in a direct comparison between real and sham, it suggests a net zero-sum scenario. This possibility, and the discrepancies between previous studies, point to the need for further careful investigation.

In conclusion, there was no significant group difference in cognitive performance after iTBS of the left DLPFC in subjects with PD-MCI during a double-blind, randomized, and sham-controlled trial. Stimulation modulated functional connectivity between the stimulation site and the ipsilateral caudate head, which was observed 48 h after the last stimulation session. Finally, higher baseline connectivity between the stimulation network and the striatal network was associated with a greater change in executive functioning in the real stimulation group. This study supports further investigation of iTBS in PD-MCI for the treatment of cognitive impairment. Future trials should focus on executive functioning abilities and should incorporate delayed outcome measurements. Stimulation targeting could be guided by baseline connectivity in corticostriatal networks.

## Data Availability Statement

The datasets presented in this article are not readily available because the data that support the finding of this article are available from the corresponding author, upon request. Requests to access the datasets should be directed to Oury Monchi, oury.monchi@ucalgary.ca.

## Ethics Statement

The studies involving human participants were reviewed and approved by University of Calgary Research Ethics Board. The patients/participants provided their written informed consent to participate in this study.

## Author Contributions

SL: conceptualization, methodology, investigation, formal analysis, writing-original draft, writing-review and editing, and visualization. LG: methodology, investigation, writing-review, and editing. EY: methodology, investigation, and formal analysis. AH: methodology and investigation. MK: investigation, methodology, data curation, writing-review, and editing. JC: investigation, project administration, and data curation. TH: investigation and data curation. IK: investigation, project administration, data curation, writing-review, and editing. JS: investigation. DM: investigation, writing-review, and editing. OM: conceptualization, methodology, writing- review and editing, and supervision. All authors contributed to the article and approved the submitted version.

## Conflict of Interest

The authors declare that the research was conducted in the absence of any commercial or financial relationships that could be construed as a potential conflict of interest.

## Abbreviations

PD-MCI: Parkinson's disease with mild cognitive impairment
DLPFC: dorsolateral prefrontal cortex
TMS: transcranial magnetic stimulation
rTMS: repetitive transcranial magnetic stimulation
iTBS: intermittent theta-burst stimulation
UPDRS-III: Unified Parkinson's disease rating scale part III
BDI-II: Beck Depression Inventory II
BAI: Beck Anxiety Inventory
aMT: active motor threshold
ROI: region of interest
BOLD: blood oxygen level-dependent
CSF: cerebrospinal fluid
MoCA: Montreal Cognitive Assessment.
