# Supplementary material for: Theta-Burst Stimulation for Cognitive Enhancement in Parkinson's Disease With Mild Cognitive Impairment: A Randomized, Double-Blind, Sham-Controlled Trial
Source: Front Neurol. 2020 Dec 21;11:584374. doi: 10.3389/fneur.2020.584374 (PMC7779796; doi:10.3389/fneur.2020.584374)
Supplement: Supplementary file 1 [file Table_1.docx]

**Theta-burst stimulation for cognitive enhancement in Parkinson’s disease with mild cognitive impairment: a randomized, double-blind, sham-controlled trial**

Stefan Lang MD^1,2,4^, Liu Shi Gan PhD^1,4^, Eun Jin Yoon PhD^1^, Alexandru Hanganu MD, PhD^1,2,5^, Mekale Kibreab BA^1^, Jenelle Cheetham BSc^1^, Tracy Hammer RN^1^, Iris Kathol PhD^1^, Justyna Sarna MD, PhD^1,2^, Davide Martino MD, PhD^1,2,4^, Oury Monchi PhD ^1,2,3,4^

1 Cumming School of Medicine, Hotchkiss Brain Institute, Calgary, AB, CA

2 Department of Clinical Neurosciences, University of Calgary, AB, CA

3 Department of Radiology, University of Calgary, Calgary, AB, CA

4 Non-invasive Neurostimulation Network, University of Calgary, AB, CA

5 Institut Universitaire de Gériatrie de Montréal, Centre de Recherche, Montreal, QC, CA

**Supplementary Table I. Reasons for exclusion**

**Reasons for Exclusion:**

40 Non-MCI

12 Age criteria

16 PD diagnostic criteria

09 Prior stroke

09 DBS

02 Psychiatric disorders

03 Dementia

20 Medical conditions

12 MRI exclusions

13 Personal reasons

33 Unknown
