# Supplementary material for: Theta-Burst Stimulation for Cognitive Enhancement in Parkinson's Disease With Mild Cognitive Impairment: A Randomized, Double-Blind, Sham-Controlled Trial
Source: Front Neurol. 2020 Dec 21;11:584374. doi: 10.3389/fneur.2020.584374 (PMC7779796; doi:10.3389/fneur.2020.584374)
Supplement: Supplementary file 2 [file Table_2.docx]

**Theta-burst stimulation for cognitive enhancement in Parkinson’s disease with mild cognitive impairment: a randomized, double-blind, sham-controlled trial**

Stefan Lang MD^1,2,4^, Liu Shi Gan PhD^1,4^, Eun Jin Yoon PhD^1^, Alexandru Hanganu MD, PhD^1,2,5^, Mekale Kibreab BA^1^, Jenelle Cheetham BSc^1^, Tracy Hammer RN^1^, Iris Kathol PhD^1^, Justyna Sarna MD, PhD^1,2^, Davide Martino MD, PhD^1,2,4^, Oury Monchi PhD ^1,2,3,4^

1 Cumming School of Medicine, Hotchkiss Brain Institute, Calgary, AB, CA

2 Department of Clinical Neurosciences, University of Calgary, AB, CA

3 Department of Radiology, University of Calgary, Calgary, AB, CA

4 Non-invasive Neurostimulation Network, University of Calgary, AB, CA

5 Institut Universitaire de Gériatrie de Montréal, Centre de Recherche, Montreal, QC, CA

**Supplementary Table 2:** **Neuropsychological Tests grouped into five cognitive domains**

| **Executive Function** |  |
| --- | --- |
|  | Stroop Color and Word Test* |
|  | Brixton Spatial Anticipation* |
|  | Hayling Sentence Completion Section 2 |
|  | Trail Making Test (B) |
|  | Clock Drawing Test (Command) |
| **Attention** |  |
|  | Trail Making Test (A) |
|  | WMS-IV Symbol Span* |
|  | WAIS-IV Digit Span (Forward) |
| **Language** |  |
|  | Boston Naming Test |
|  | Semantic Fluency (Animals/Actions)* |
| **Visuospatial** |  |
|  | Benton JOLO |
|  | RCFT copy trial |
| **Memory** |  |
|  | HVLT (Retention/Discrimination Index) |
|  | WMS-IV Logical Memory |
|  | RCFT delayed recall trial |

WMS = Wechsler Memory Scale; WAIS = Wechsler Adult Intelligence Scale; JOLO: Judgement of Line Orientation; RCFT: Rey Complex Figure Copy Test; HVLT: Hopkins Verbal Learning Test

*No alternative forms available. All other tests had alternative forms for each follow-up assessment.
