# Supplementary material for: Theta-Burst Stimulation for Cognitive Enhancement in Parkinson's Disease With Mild Cognitive Impairment: A Randomized, Double-Blind, Sham-Controlled Trial
Source: Front Neurol. 2020 Dec 21;11:584374. doi: 10.3389/fneur.2020.584374 (PMC7779796; doi:10.3389/fneur.2020.584374)
Supplement: Supplementary file 3 [file Table_3.docx]

**Theta-burst stimulation for cognitive enhancement in Parkinson’s disease with mild cognitive impairment: a randomized, double-blind, sham-controlled trial**

Stefan Lang MD^1,2,4^, Liu Shi Gan PhD^1,4^, Eun Jin Yoon PhD^1^, Alexandru Hanganu MD, PhD^1,2,5^, Mekale Kibreab BA^1^, Jenelle Cheetham BSc^1^, Tracy Hammer RN^1^, Iris Kathol PhD^1^, Justyna Sarna MD, PhD^1,2^, Davide Martino MD, PhD^1,2,4^, Oury Monchi PhD ^1,2,3,4^

1 Cumming School of Medicine, Hotchkiss Brain Institute, Calgary, AB, CA

2 Department of Clinical Neurosciences, University of Calgary, AB, CA

3 Department of Radiology, University of Calgary, Calgary, AB, CA

4 Non-invasive Neurostimulation Network, University of Calgary, AB, CA

5 Institut Universitaire de Gériatrie de Montréal, Centre de Recherche, Montreal, QC, CA

**Supplementary Table 3 - Adverse Events**

| **Relation to Study** | **Incident** | **Action Taken/Outcome** |
| --- | --- | --- |
| Definitely Related | Participant had uncomfortable sensation over left eye/forehead area during iTBS application. | Stimulation intensity was reduced by 3% at which point the uncomfortable sensation resolved. |
| Definitely Related | Participant had uncomfortable sensation over stimulation site area during iTBS application. | Stimulation intensity was reduced by 5% at which point the uncomfortable sensation resolved. |
| Definitely Related | Participant had uncomfortable sensation over stimulation site/left forehead area during iTBS application. | Stimulation intensity was reduced by 3% at which point the uncomfortable sensation resolved. |
| Definitely Related | Participant had uncomfortable sensation over stimulation site area during iTBS application. | Stimulation intensity was reduced by 5% at which point the uncomfortable sensation resolved. |
| Probably Related | Participant had uncomfortable sensation in tooth during iTBS application | Symptoms resolved spontaneously and stimulation continued as per protocol. |
| Possibly Related | Participant experienced discomfort (not painful) over left eye following iTBS application. This lasted for several days. | Participant remained in the study and continued as per protocol. Symptoms resolved spontaneously. |
| Possibly Related | Participant experienced minor headache following the first iTBS application. | Participant remained in the study and continued as per protocol. Symptoms resolved spontaneously. |
| No Relation | Participant had neurological event (Transient Ischemic Attack) while enrolled in the study, resulting in hospitalization. This did not occur during any of the laboratory visits. | Participant was discontinued from the study. |
