# Supplementary material for: Theta-Burst Stimulation for Cognitive Enhancement in Parkinson's Disease With Mild Cognitive Impairment: A Randomized, Double-Blind, Sham-Controlled Trial
Source: Front Neurol. 2020 Dec 21;11:584374. doi: 10.3389/fneur.2020.584374 (PMC7779796; doi:10.3389/fneur.2020.584374)
Supplement: Supplementary file 4 [file Table_4.docx]

**Theta-burst stimulation for cognitive enhancement in Parkinson’s disease with mild cognitive impairment: a randomized, double-blind, sham-controlled trial**

Stefan Lang MD^1,2,4^, Liu Shi Gan PhD^1,4^, Eun Jin Yoon PhD^1^, Alexandru Hanganu MD, PhD^1,2,5^, Mekale Kibreab BA^1^, Jenelle Cheetham BSc^1^, Tracy Hammer RN^1^, Iris Kathol PhD^1^, Justyna Sarna MD, PhD^1,2^, Davide Martino MD, PhD^1,2,4^, Oury Monchi PhD ^1,2,3,4^

1 Cumming School of Medicine, Hotchkiss Brain Institute, Calgary, AB, CA

2 Department of Clinical Neurosciences, University of Calgary, AB, CA

3 Department of Radiology, University of Calgary, Calgary, AB, CA

4 Non-invasive Neurostimulation Network, University of Calgary, AB, CA

5 Institut Universitaire de Gériatrie de Montréal, Centre de Recherche, Montreal, QC, CA

**Supplementary Table 4: Cognitive scores across time**

| **Primary Outcomes** | **Real: mean (SD)** | **Sham: mean (SD)** | **Main effect: Condition** | **Main effect: Early** | **Main effect: Late** | **Interaction: Early*condition** | **Interaction: Late*condition** |
| --- | --- | --- | --- | --- | --- | --- | --- |
|  |  |  |  |  |  |  |  |
| **Global Cognition** |  |  |  |  |  |  |  |
| **Pre** | -0.62 (0.51) | -0.72 (0.61) | ns | * | * | ns | ns |
| **Early** | -0.56 (0.52) | -0.56 (0.68) |  | | | | |
| **Late** | -0.41 (0.63) | -0.48 (0.71) |  |  |  |  |  |
| **Executive Function** |  |  |  |  |  |  |  |
| **Pre** | -0.87 (0.66) | -0.71 (0.93) | ns | ns | ns | ns | * |
| **Early** | -0.47 (0.65) | -0.54 (0.86) |  | | | | |
| **Late** | -0.45 (0.85) | -0.56 (0.92) |  |  |  |  |  |
| **Language** |  |  |  |  |  |  |  |
| **Pre** | -0.47 (0.83) | -0.51 (0.77) | ns | * | ns | ns | ns |
| **Early** | -0.46 (0.75) | -0.21 (0.89) |  |  |  |  |  |
| **Late** | -0.38 (0.79) | -0.29 (0.91) |  |  |  |  |  |
| **Attention** |  |  |  |  |  |  |  |
| **Pre** | -0.50 (0.45) | -0.50 (0.53) | ns | ns | ns | ns | ns |
| **Early** | -0.39 (0.61) | -0.38 (0.77) |  | | | | |
| **Late** | -0.35 (0.56) | -0.33 (0.87) |  |  |  |  |  |
| **Memory** |  |  |  |  |  |  |  |
| **Pre** | -0.52 (0.75) | -0.64 (0.78) | ns | ns | ns | ns | ns |
| **Early** | -0.74 (0.75) | -0.61 (0.97) |  | | | | |
| **Late** | -0.31 (0.82) | -0.39 (0.92) |  |  |  |  |  |
| **Visuospatial** |  |  |  |  |  |  |  |
| **Pre** | -0.72 (0.81) | -1.27 (0.85) | * | ns | * | ns | ns |
| **Early** | -0.75 (0.78) | -1.10 (0.86) |  | | | | |
| **Late** | -0.59 (0.93) | -0.83 (0.84) |  |  |  |  |  |

SD = standard deviation; * = p<0.05 uncorrected; ns = not significant(p>0.05); Early = 24 hours post-stimulation; Late = 1 month post-stimulation
