# Supplementary material for: Theta-Burst Stimulation for Cognitive Enhancement in Parkinson's Disease With Mild Cognitive Impairment: A Randomized, Double-Blind, Sham-Controlled Trial
Source: Front Neurol. 2020 Dec 21;11:584374. doi: 10.3389/fneur.2020.584374 (PMC7779796; doi:10.3389/fneur.2020.584374)
Supplement: Supplementary file 5 [file Data_Sheet_1.zip › Supplementary Methods I.DOCX]

**Theta-burst stimulation for cognitive enhancement in Parkinson’s disease with mild cognitive impairment: a randomized, double-blind, sham-controlled trial**

Stefan Lang MD^1,2,4^, Liu Shi Gan PhD^1,4^, Eun Jin Yoon PhD^1^, Alexandru Hanganu MD, PhD^1,2,5^, Mekale Kibreab BA^1^, Jenelle Cheetham BSc^1^, Tracy Hammer RN^1^, Iris Kathol PhD^1^, Justyna Sarna MD, PhD^1,2^, Davide Martino MD, PhD^1,2,4^, Oury Monchi PhD ^1,2,3,4,5^

1 Cumming School of Medicine, Hotchkiss Brain Institute, Calgary, AB, CA

2 Department of Clinical Neurosciences, University of Calgary, AB, CA

3 Department of Radiology, University of Calgary, Calgary, AB, CA

4 Non-invasive Neurostimulation Network, University of Calgary, AB, CA

5 Institut Universitaire de Gériatrie de Montréal, Centre de Recherche, Montreal, QC, CA

**Supplementary Methods I**

**Stimulation Network**

The stimulation network was defined in an independent sample of 60 PD subjects by assessing the whole brain connectivity of the stimulation site and extracting highly significant clusters (one sample t-test, p<1*10^-10^, p<0.01 cluster FDR correction) to be used as ROI’s. MRI acquisition and preprocessing were identical to that described in Supplementary Methods IV. This resulted in seven cortical ROIs.

**Stimulation Network Regions of Interest**

| **Region** | **Central MNI coordinates (x,y,z)** | **Size (voxel #)** |
| --- | --- | --- |
| Left Mid Frontal Gyrus | -46 +28 +38 | 5291 |
| Left Inferior Parietal Gyrus | -47, -57, 47 | 2256 |
| Right Mid Frontal Gyrus | +47 +27 +38 | 1405 |
| Right Angular Gyrus | +43, -65, 50 | 705 |
| Precuneus | -3, -70, 47 | 162 |
| Right Superior Frontal Gyrus | +23 +24 +50 | 142 |
| Frontal Superior Medial Gyrus | -1, 27, 46 | 1061 |

Region description based on central MNI coordinates correspondence with the Automated Anatomical Labelling atlas
