# Supplementary material for: Theta-Burst Stimulation for Cognitive Enhancement in Parkinson's Disease With Mild Cognitive Impairment: A Randomized, Double-Blind, Sham-Controlled Trial
Source: Front Neurol. 2020 Dec 21;11:584374. doi: 10.3389/fneur.2020.584374 (PMC7779796; doi:10.3389/fneur.2020.584374)
Supplement: Supplementary file 5 [file Data_Sheet_1.zip › Supplementary Methods II.DOCX]

**Theta-burst stimulation for cognitive enhancement in Parkinson’s disease with mild cognitive impairment: a randomized, double-blind, sham-controlled trial**

Stefan Lang MD^1,2,4^, Liu Shi Gan PhD^1,4^, Eun Jin Yoon PhD^1^, Alexandru Hanganu MD, PhD^1,2,5^, Mekale Kibreab BA^1^, Jenelle Cheetham BSc^1^, Tracy Hammer RN^1^, Iris Kathol PhD^1^, Justyna Sarna MD, PhD^1,2^, Davide Martino MD, PhD^1,2,4^, Oury Monchi PhD ^1,2,3,4,5^

1 Cumming School of Medicine, Hotchkiss Brain Institute, Calgary, AB, CA

2 Department of Clinical Neurosciences, University of Calgary, AB, CA

3 Department of Radiology, University of Calgary, Calgary, AB, CA

4 Non-invasive Neurostimulation Network, University of Calgary, AB, CA

5 Institut Universitaire de Gériatrie de Montréal, Centre de Recherche, Montreal, QC, CA

**Supplementary Methods II**

**Modified MCI criteria**

We repeated the analysis of executive function scores using only subjects who met MCI criteria with less than 1.5SD in any two tests. This resulted in the exclusion of 12 subjects. The remaining 29 (15 real, 14 sham) were reanalyzed. There was no significant baseline, main, or interaction effect using this modified cohort of subjects.

Next, we assessed whether there was any relationship between baseline global cognitive or baseline executive functioning scores and the observed change in executive function at the delayed timepoint in the real stimulation group. This was performed with a correlation analysis, with the change in executive function as the dependent variable, and baseline scores as the independent variable. We did not observe a significant relationship between baseline global cognition and change in executive function (r = 0.373, p = 0.0957), nor between baseline executive function and the change in executive function (r = 0.0361, p = 0.8764).

Given the uneven and small number of subjects within each cognitive impairment subtype, we did not analyze these subgroups separately.

| **Cognitive Impairment Subtype** | **Number of Subjects** |
| --- | --- |
| Single domain amnestic | 2 (4.8%) |
| Multi-domain amnestic | 25 (61%) |
| Single domain non-amnestic | 1 (2.4%) |
| Multi-domain non-amnestic | 13 (31.7%) |
